# Bearing Damage Detection of a Reinforced Concrete Plate Based on Sensitivity Analysis and Chaotic Moth-Flame-Invasive Weed Optimization

**DOI:** 10.3390/s20195488

**Published:** 2020-09-25

**Authors:** Minshui Huang, Yongzhi Lei

**Affiliations:** School of Civil Engineering and Architecture, Wuhan Institute of Technology, Wuhan 430073, China; mr_lei.yz@wit.edu.cn

**Keywords:** damage detection, bearing damage, sensitivity analysis, regularization technology, moth-flame optimization, invasive weed, hybrid optimization

## Abstract

This article proposes a novel damage detection method based on the sensitivity analysis and chaotic moth-flame-invasive weed optimization (CMF-IWO), which is utilized to simultaneously identify the damage of structural elements and bearings. First, the sensitivity coefficients of eigenvalues to the damage factors of structural elements and bearings are deduced, the regularization technology is used to solve the problem of equation undetermined, meanwhile, the modal strain energy-based index is utilized to detect the damage locations, and the regularization objective function is constructed to quantify the damage severity. Then, for the subsequent procedure of damage detection, CMF-IWO is proposed based on moth-flame optimization and invasive weed optimization as well as chaos theory, reverse learning, and evolutional strategy. The optimization effectiveness of the hybrid algorithm is verified by five benchmark functions and a damage identification numerical example of a simply supported beam; the results demonstrate it is of great global search ability and higher convergence efficiency. After that, a numerical example of an 8-span continuous beam and an experimental reinforced concrete plate are both adopted to evaluate the proposed damage identification method. The results of the numerical example indicate that the proposed method can locate and quantify the damage of structural elements and bearings with high accuracy. Furthermore, the outcomes of the experimental example show that despite the existence of some errors and uncertain factors, the method still obtains an acceptable result. Generally speaking, the proposed method is proved that it is of good feasibility.

## 1. Introduction

Civil structures suffer from traffic load, environmental temperature variation, fatigue failure, and other uncertain negative influences during the service period. Among the distinguished damage identification methods, the modal parameters-based detection method is known as the most popular one. There are some major reasons for understanding its popularity. First, the modal parameters, such as natural frequencies and modal shapes, are obtained easily by modal tests. Meanwhile, prior studies have addressed many issues, the common one of them is the sensitivity coefficient analysis.

The existing studies have provided several different sensitivity coefficient analyses, including the natural frequencies [1], modal shapes [2], modal strain energy [3], and modal kinetic energy [4,5]. For the natural frequencies, Xia and Hao [6] proposed a method of statistical damage identification based on the frequencies sensitivity analysis and finite element model updating, which was successfully applied in a numerical cantilever beam and a laboratory tested steel cantilever plate, meanwhile, the number of modal orders, various noise level, and different damage severity were also investigated. In the study of Kim et al. [7], they evaluated and compared the sensitivity methods of natural frequencies and modal shapes using a numerical concrete beam; results showed both methods could localize and estimate the damage. Furthermore, Wei et al. [8] localized damage using the modal strain energy change ratio approach, and good identification results in numerical thin plates were obtained. Dinh-Cong et al. [9] derived the sensitivity coefficient of modal kinetic energy change ratio (MKECR), then combined with symbiotic organisms search for damage localization and quantification, the research indicated MKECR with good capability in locating the large numbers of damage locations despite the existence of measurement noise.

However, because the dimensions of measured modal information are far less than the degree of freedoms of the structure, the methods of sensitivity analyses have to face the problem of equation underdetermined [10]. This issue will cause infinite solutions, which can be overcome by adopting the regularization technology. Following this idea, Zhou et al. [11] achieved the damage detection of the experimental cantilever beam based on the frequencies sensitivity analysis and *l*_1_ regularization approach. The most significant advantage in the study was that only the first few structural frequencies were used. Then, for the purpose of enhancing the accuracy, Hou et al. [12] utilized the modal shapes, and the selection of regularization parameter was also studied [13], the results of numerical and experimental examples indicated that the method could localize and estimate the sparse damage over a numerous number of structural elements.

The damage identification based on the modal parameters requires the process of iterative computation, since it is always transformed into a problem of mathematical optimization. However, this process will confront another disturbing problem, namely, low computational efficiency and local optimal. Hence, some swarm intelligence and evolutionary algorithms are exploited to tackle the difficulty. One of the most popular is genetic algorithm (GA) [14]. In fact, there exist certain challenges when practicing GA. At first, although there are numerous applications of GA [15,16,17], it is not convenient to apply because of its complexity of parameter initialization. Also, GA tends to be trapped into local optimal, which causes the low inaccuracy of damage identification. Aiming to these drawbacks, some other optimization tools, such as Particle Swarm Optimization (PSO) [18], Cuckoo Search (CS) [19], Jaya algorithm [20], Artificial Fish Swarm Optimization (AFSWO) [21], and Artificial Bee Colony algorithm [22], were adopted in previous studies as well. Furthermore, based on the hybrid mechanism of different algorithms, better optimization performance can be achieved. For example, Huang et al. [23] proposed a PSO-CS hybrid algorithm. The random elimination mechanism of CS was incorporated into basic PSO to avoid trapped in a local optimum. Ding et al. [24] combined the Jaya and Tree Seeds algorithm to consider the uncertainties in structural damage identification.

On the other hand, for the damage detection of structural bearings, the existing researches usually concern about the identification of boundary conditions. For example, Akhtyamov and Mouftakhov [25] used the first four natural frequencies to identify the elastic constraint on each side of the rectangular membrane. Then, Chesne [26] illustrated a method based on the estimation of the spatial derivatives of displacement, which can be used to determine the boundary conditions of a beam. Ahmadian and Esfandiar [27] adopted natural frequencies and damping ratios to estimate the boundary condition parameters of a rectangular plate restrained in edges by an elastic support. In the paper of Wang and Yang [28], a direct method was reported to identify the boundary conditions of tapered beam-like structures using static flexibility measurements. Furthermore, there still exist some papers that pay attention to the influence of boundary conditions. Xia et al. [29] proposed a novel approach to perform the automatic reconstruction of boundary conditions in structure analysis, which was devoted to improve the mechanical design efficiency. Park et al. [30] tried to use neural networks to consider the boundary conditions in the process of finite element model updating. Shi et al. [31] also studied the problem of updating boundary conditions for bridge structures.

Despite many studies on the structural damage and boundary condition identification, little to no researches, to our best knowledge, focus on the bearings damage detection of beam structures, even to detect a beam structure whose damage occurs in structural elements and bearings simultaneously. At the same time, the existing methods are too complex to obtain the results of boundary conditions, which are not easy to be conducted in the practical applications. However, the rubber bearing, known as one of the most important components of a beam structure, connects the beam and the pier or abutment. The bearing is easy to be damaged in its complicated service environment. The common diseases include separation, shear deformation, bulging deformation, and transverse crack. To address this problem, Ni et al. [32] proposed a damage index for bridge pot rubber bearing based on support vector machine and correlation and sensitivity analysis. Chen et al. [33] used the radial basis function neural network and combined with bridge vibration modes to identify the bridge bearing damage, but the damage severity failed to be estimated. The existence of bearings diseases will alter the forced form of the whole bridge. In a worse situation, the bridge girder and pier would be damaged as well. Thus, the damage of the bearing should be regarded as a potential risk in shortening the life cycle of bridges. As a result, there would be a great demand for promoting an easy and convenient method to detect the damage location and severity of bearings.

In this paper, a novel damage detection method is proposed to simultaneously identify the damage of structural elements and bearings. Based on this method, satisfactory results can be achieved by using only several orders modal characteristics, and complicated calculations in the existing methods can be avoided. The main significance of this study can be listed as follows: (1) The sensitivity coefficients of eigenvalues to the damage factors of structural elements and bearings are deduced, which is a creative theory that can be used to identify the damage of bearings; (2) a hybrid algorithm, chaotic moth-flame-invasive weed optimization (CMF-IWO), is raised to improve the optimization problem, such as local optimal and slow convergence and it is proved that CMF-IWO has better computational performance than other commonly used algorithms; (3) based on the first few modal characteristics, the proposed method not only identify structural damage but also determine the damage of bearings, which is the first damage identification method that can simultaneously consider the damage of structures and bearings.

The specific work can be listed as follows: At first, the sensitivity coefficients of eigenvalues to the damage factors of structural elements and bearings are derived, which can simultaneously consider the influence of the damage of structural elements and bearings on eigenvalues. Meanwhile, based on the sensitivity analysis, regularization objective function is constructed to detect the damage of structure and bearings; modal strain energy-based index (MSEBI) is utilized to locate damage location. Then, a new hybrid optimization algorithm, named as chaotic moth-flame-invasive weed optimization (CMF-IWO), is proposed on the basis of moth-flame optimization (MFO), invasive weed optimization (IWO), and some enhanced techniques, such as chaos theory, reverse learning and evolutional strategy. Five benchmark functions and a numerical damage identification example are adopted to make comparisons with PSO, CS, MFO, Differential Evolution (DE), and IWO. The optimization results and iterative curves show that CMF-IWO is of better optimization capability. After that, a numerical 8-span continuous beam and an experimental simply supported reinforced concrete plate both are used to verify the feasibility of the proposed method. The outcomes demonstrate that the proposed method can accurately localize and quantify the damage of elements and bearings in the numerical example. Though there exist some uncertain factors for the experimental, the method has been proved to detect the separation of bearing with high accuracy. In addition, considering the practical situation, there are some acceptable errors in the damage quantification.

## 2. Damage Detection Model

### 2.1. Sensitivity Analysis on the Damage of Structural Elements and Bearings

#### 2.1.1. Damage Description of Structural Elements and Bearings

According to the theories of structural dynamics and finite element method, the un-damped free vibration differential equation of a structure can be written as follows:

(1) $$(K-\lambda_{i}M)\varphi_{i}=0$$

where K and M denote the global stiffness and mass matrices of a structure, respectively; $\lambda_{i}$ and $\varphi_{i}$ indicate the *i*-th eigenvalue and eigenvector, respectively. Very few researches try to establish a model with structural boundary condition, and it is only treated as a simple constraint. However, this issue can be solved by dividing the structure into two systems, one is the structural system and the other is the boundary condition system [1]. Thus, Equation (1) can be rewritten as follows:

(2) $$[(K_{s}+K_{bc})-\lambda_{i}(M_{s}+M_{bc})]\varphi_{i}=0$$

where $K_{s}$ and $K_{bc}$ mean the stiffness matrices of the structural and boundary condition system, respectively; $M_{s}$ and $M_{bc}$ stand for the mass matrices of the structural system and boundary condition system, respectively. Also, the stiffness matrix of the boundary condition system can be defined as follows [1]:

(3) $$\begin{array}{l} \;Ux\;Rx\;Uy\;Ry\;Uz\;Rz\\ \begin{array}{c} Ux\\ Rx\\ Uy\\ Ry\\ Uz\\ Rz \end{array}\left[\begin{array}{cccccc} A & & & B & & C\\ & D & E & & F & \\ & & G & & & H\\ & Sym & & I & J & \\ & & & & K & \\ & & & & & L \end{array}\right] \end{array}$$

where the elements on the diagonal of the matrix denote the boundary stiffness in every degree of freedom (DOF); others mean the synergistic effect, which can be explained that the translation in one direction will cause rotations in the other two directions. Meanwhile, this matrix is not only available for a 3-D finite element model, but also can be applied for the 2-D model by reducing the unrelated DOFs.

When the damage of structural elements and/or bearings arise in the structure, damage can be seen as the stiffness reduction with no mass change, thus, the damage of structural elements and bearings can be quantitatively measured by using stiffness reduction vectors:

(4) $$\theta =[\theta_{1},\theta_{2},\theta_{3},\cdots ,\theta_{k}]$$

(5) $$\alpha =[\alpha_{1},\alpha_{2},\alpha_{3},\cdots ,\alpha_{j}]$$

where $\theta_{k}$ and $\alpha_{j}$ represent the damage severity of the *k*-th element and the *j*-th bearing. Thereby, the damage in structural element and bearing can be described as follows:

(6) $$K_{s}=\sum_{k=1}^{nele}(1-\theta_{k})k_{k}^{s},0\leq \theta_{k}\leq 1$$

(7) $$K_{bc}=\sum_{j=1}^{nbc}(1-\alpha_{j})k_{j}^{bc},0\leq \alpha_{j}\leq 1$$

where *nele* is the number of the structural elements; *nbc* is the number of bearings.

#### 2.1.2. Sensitivity Analysis of Eigenvalue

The first-order sensitivity coefficients of eigenvalue corresponding to the structural damage factor $\theta_{k}$ and the bearing damage factor $\alpha_{j}$ can be obtained by derivative of Equation (2) concerning the two parameters, respectively, which can be written as follows:

(8) $$[\frac{\partial }{\partial \theta_{k}}(K_{s}+K_{bc})-\frac{\partial \lambda_{i}}{\partial \theta_{k}}(M_{s}+M_{bc})-\lambda_{i}\frac{\partial }{\partial \theta_{k}}(M_{s}+M_{bc})]\varphi_{i}+[(K_{s}+K_{bc})-\lambda_{i}(M_{s}+M_{bc})]\frac{\partial \varphi_{i}}{\partial \theta_{k}}=0$$

(9) $$[\frac{\partial }{\partial \alpha_{j}}(K_{s}+K_{bc})-\frac{\partial \lambda_{i}}{\partial \alpha_{j}}(M_{s}+M_{bc})-\lambda_{i}\frac{\partial }{\partial \alpha_{j}}(M_{s}+M_{bc})]\varphi_{i}+[(K_{s}+K_{bc})-\lambda_{i}(M_{s}+M_{bc})]\frac{\partial \varphi_{i}}{\partial \alpha_{j}}=0$$

Then, the above equations are premultiplied by $\varphi_{i}^{T}$ in both sides. At the same time, using the known relations, such as $\varphi_{i}^{T}(M_{s}+M_{bc})\varphi_{i}=I$ for the unit-mass normalized mode shapes; the mass matrices $M_{s}$ and $M_{bc}$ are independent of the damage factors of structural elements and bearings; stiffness matrices $K_{s}$ and $K_{bc}$ are respectively independent of $\alpha_{j}$ and $\theta_{k}$, namely, $\partial M_{s}/\partial \theta_{k}=0$, $\partial M_{bc}/\partial \alpha_{j}=0$, $\partial K_{bc}/\partial \theta_{k}=0$ and $\partial K_{s}/\partial \alpha_{j}=0$. Thus, the sensitivity coefficients of *i*-th eigenvalue corresponding to $\theta_{k}$ and $\alpha_{j}$ can be derived respectively as follows:

(10) $$\frac{\partial \lambda_{i}}{\partial \theta_{k}}=\varphi_{i}^{T}\frac{\partial K_{s}}{\partial \theta_{k}}\varphi_{i}$$

(11) $$\frac{\partial \lambda_{i}}{\partial \alpha_{j}}=\varphi_{i}^{T}\frac{\partial K_{bc}}{\partial \alpha_{j}}\varphi_{i}$$

According to Equations (6) and (7), the following equations can be derived as:

(12) $$\frac{\partial K_{s}}{\partial \theta_{k}}=\frac{\partial (\sum_{k=1}^{nele}\left(1-\theta_{k})k_{k}^{s}\right)}{\partial \theta_{k}}=-K_{k}^{s}$$

(13) $$\frac{\partial K_{bc}}{\partial \alpha_{j}}=\frac{\partial (\sum_{i=1}^{nbc}\left(1-\alpha_{j})k_{j}^{bc}\right)}{\partial \alpha_{j}}=-K_{j}^{bc}$$

where $K_{k}^{s}$ and $K_{j}^{bc}$ denote the *k*-th structural element stiffness matrix and the *j*-th boundary condition stiffness matrix in the global coordinate, respectively. Thus, the first-order sensitivity coefficients of eigenvalue corresponding to $\theta_{k}$ and $\alpha_{j}$ can be rewritten as follows:

(14) $$S_{\lambda }^{s}=\frac{\partial \lambda_{i}}{\partial \theta_{k}}=-\varphi_{i}^{T}K_{k}^{s}\varphi_{i}$$

(15) $$S_{\lambda }^{bc}=\frac{\partial \lambda_{i}}{\partial \alpha_{j}}=-\varphi_{i}^{T}K_{j}^{bc}\varphi_{i}$$

#### 2.1.3. Sensitivity Analysis of Eigenvector

According to the paper of Zhao and DeWolf [34], the first-order sensitivity coefficient of the eigenvector corresponding to the structural damage factor $\theta_{k}$ can be represented as follows:

(16) $$\frac{\partial \varphi_{i}}{\partial \theta_{k}}=\sum_{n=1}^{ndof}a_{in}\varphi_{n}$$

where *ndof* stands for the total number of structural DOFs; $a_{in}$ denotes the *n*-th undetermined coefficient in the sensitivity coefficient of the *i*-th eigenvector to the structural damage factor $\theta_{k}$. As for $a_{in}$, there are two possible situations:

(1) When the subscript i≠n, Equation (8) is premultiplied by $\varphi_{n}^{T}$ on its both sides, then substituting Equation (16) into Equation (8), the following equation can be derived:
  (17) $$\varphi_{n}^{T}[\frac{\partial }{\partial \theta_{k}}(K_{s}+K_{bc})-\frac{\partial \lambda_{i}}{\partial \theta_{k}}(M_{s}+M_{bc})-\lambda_{i}\frac{\partial }{\partial \theta_{k}}(M_{s}+M_{bc})]\varphi_{i}+\varphi_{n}^{T}[(K_{s}+K_{bc})-\lambda_{i}(M_{s}+M_{bc})]\sum_{n=1}^{ndof}a_{in}\varphi_{n}=0$$
  Because of the orthogonality of mode shapes, the mathematical relation can be concluded, namely, if i≠n, $\varphi_{n}^{T}(M_{s}+M_{bc})\varphi_{i}=0$; $\varphi_{n}^{T}(M_{s}+M_{bc})\varphi_{n}=I$; $\varphi_{n}^{T}(K_{s}+K_{bc})=\lambda_{n}\varphi_{n}^{T}(M_{s}+M_{bc})$; and $\partial M_{s}/\partial \theta_{k}=0$. Thus, Equation (17) can be rewritten as follows:
  (18) $$-\varphi_{n}^{T}K_{s}\varphi_{i}+(\lambda_{n}-\lambda_{i})\sum_{n=1}^{ndof}a_{in}=0$$
  For $a_{in}$, it can be solved as:
  (19) $$a_{in}=\frac{1}{\left(\lambda_{n}-\lambda_{i}\right)}\varphi_{n}^{T}K_{k}^{s}\varphi_{i}$$
(2) When the subscript i=n, as for the unit-mass normalized mode shapes, it can obtain:
  (20) $$\varphi_{n}^{T}(M_{s}+M_{bc})\varphi_{i}=I$$
  Aiming Equation (20), take derivative in terms of $\theta_{k}$, which can be written as:
  (21) $$\frac{\partial \varphi_{n}^{T}}{\partial \theta_{k}}(M_{s}+M_{bc})\varphi_{i}+\varphi_{n}^{T}(M_{s}+M_{bc})\frac{\partial \varphi_{i}}{\partial \theta_{k}}=0$$
  Noting the symmetry characteristic of the mass matrix:
  (22) $$\frac{\partial \varphi_{n}^{T}}{\partial \theta_{k}}(M_{s}+M_{bc})\varphi_{i}+\varphi_{n}^{T}(M_{s}+M_{bc})\frac{\partial \varphi_{i}}{\partial \theta_{k}}$$
  Considering Equation (16), the above equation can be rewritten as:
  (23) $$2\sum_{n=1}^{ndof}a_{in}\varphi_{n}^{T}(M_{s}+M_{bc})\varphi_{i}$$
  Because of the orthogonality of mode shapes and Equation (23), it can be indicated that:
  (24) $$a_{in}=0$$
  Based on the above analysis, the sensitivity coefficient of a mode shape corresponding to the structural element damage factor can be summarized as follows:
  (25) $$S_{\varphi }^{s}=\frac{\partial \varphi_{i}}{\partial \theta_{k}}=\{\begin{array}{cc} \sum_{n=1,n\neq i}^{ndof}\frac{1}{\left(\lambda_{n}-\lambda_{i}\right)}\varphi_{n}^{T}K_{k}^{s}\varphi_{i}\varphi_{n} & i\neq n\\ 0 & i=n \end{array}$$
  Meanwhile, the same derivation procedure may be easily adapted to obtain the sensitivity coefficient of mode shapes corresponding to bearing damage factor $\alpha_{j}$, which can be defined as:
  (26) $$S_{\varphi }^{bc}=\frac{\partial \varphi_{i}}{\partial \alpha_{j}}=\{\begin{array}{cc} \sum_{n=1,n\neq i}^{ndof}\frac{1}{\left(\lambda_{n}-\lambda_{i}\right)}\varphi_{n}^{T}K_{j}^{bc}\varphi_{i}\varphi_{n} & i\neq n\\ 0 & i=n \end{array}$$

#### 2.1.4. Sensitivity Coefficients of Eigenvalue to the Damage Factors of Structural Elements and Bearings

Because of the eigenvalues of the structure are not only influenced by the damage of structural elements but also of the bearings, therefore, the sensitivity coefficient of eigenvalue to the two factors should be considered. Based on Equations (14) and (15), the equations that can be obtained are as follows:

(27) $$\frac{\partial^{2}\lambda_{i}}{\partial \theta_{k}\partial \alpha_{j}}=\frac{\partial S_{\lambda }^{s}}{\partial \alpha_{j}}=\frac{\partial }{\partial \alpha_{j}}(-\varphi_{i}K_{k}^{s}\varphi_{i}^{T})$$

(28) $$\frac{\partial^{2}\lambda_{i}}{\partial \theta_{k}\partial \alpha_{j}}=\frac{\partial S_{\lambda }^{bc}}{\partial \theta_{k}}=\frac{\partial }{\partial \theta_{k}}(-\varphi_{i}K_{j}^{bc}\varphi_{i}^{T})$$

Expanding the above equations as:

(29) $$\frac{\partial S_{\lambda }^{s}}{\partial \alpha_{j}}=-(\frac{\partial \varphi_{i}}{\partial \alpha_{j}}K_{k}^{s}\varphi_{i}^{T}+\varphi_{i}\frac{\partial K_{s}}{\partial \alpha_{j}}\varphi_{i}^{T}+\varphi_{i}K_{k}^{s}\frac{\partial \varphi_{i}^{T}}{\partial \alpha_{j}})$$

(30) $$\frac{\partial S_{\lambda }^{bc}}{\partial \theta_{k}}=-(\frac{\partial \varphi_{i}}{\partial \theta_{k}}K_{j}^{bc}\varphi_{i}^{T}+\varphi_{i}\frac{\partial K_{bc}}{\partial \theta_{k}}\varphi_{i}^{T}+\varphi_{i}K_{j}^{bc}\frac{\partial \varphi_{i}^{T}}{\partial \theta_{k}})$$

Noting $\partial K_{bc}/\partial \theta_{k}=0$, $\partial K_{s}/\partial \alpha_{j}=0$, $S_{\lambda }^{s}=-\varphi_{i}^{T}K_{k}^{s}\varphi_{i}$ and $S_{\lambda }^{bc}=-\varphi_{i}^{T}K_{j}^{bc}\varphi_{i}$, Equations (29) and (30) are premultiplied by $\varphi_{i}^{T}$ and $\varphi_{i}$ on their right and left sides respectively:

(31) $$\varphi_{i}\frac{\partial S_{\lambda }^{s}}{\partial \alpha_{j}}\varphi_{i}^{T}=-\varphi_{i}\frac{\partial \varphi_{i}}{\partial \alpha_{j}}K_{k}^{s}{\left(\varphi_{i}^{T}\right)}^{2}-{\left(\varphi_{i}\right)}^{2}K_{k}^{s}\frac{\partial \varphi_{i}^{T}}{\partial \alpha_{j}}\varphi_{i}^{T}$$

(32) $$\varphi_{i}\frac{\partial S_{\lambda }^{bc}}{\partial \theta_{k}}\varphi_{i}^{T}=-\varphi_{i}\frac{\partial \varphi_{i}}{\partial \theta_{k}}K_{j}^{bc}{\left(\varphi_{i}^{T}\right)}^{2}-{\left(\varphi_{i}\right)}^{2}K_{j}^{bc}\frac{\partial \varphi_{i}^{T}}{\partial \theta_{k}}\varphi_{i}^{T}$$

Then the equations can be simplified by shifting terms:

(33) $$\frac{\partial S_{\lambda }^{s}}{\partial \alpha_{j}}=S_{\lambda }^{s}[\frac{\partial \varphi_{i}}{\partial \alpha_{j}}\frac{1}{\varphi_{i}}+\frac{\partial \varphi_{i}^{T}}{\partial \alpha_{j}}\frac{1}{\varphi_{i}^{T}}]$$

(34) $$\frac{\partial S_{\lambda }^{bc}}{\partial \theta_{k}}=S_{\lambda }^{bc}(\frac{\partial \varphi_{i}}{\partial \theta_{k}}\frac{1}{\varphi_{i}}+\frac{\partial \varphi_{i}^{T}}{\partial \theta_{k}}\frac{1}{\varphi_{i}^{T}})$$

Assuming $\varphi_{i}^{T}=A\theta$, then ${\left(\varphi_{i}^{T}\right)}^{T}=\varphi_{i}={\left(A\theta \right)}^{T}=\theta^{T}A^{T}$; since $\frac{\partial (A\theta )}{\partial \theta_{k}}=A^{T}$, then $\frac{\partial {\left(A\theta \right)}^{T}}{\partial \theta_{k}}=\frac{\partial (\theta^{T}A^{T})}{\partial \theta_{k}}=A$, namely, $\frac{\partial \varphi_{i}^{T}}{\partial \theta_{k}}=A^{T}$ and $\frac{\partial \varphi_{i}}{\partial \theta_{k}}=A$, thus $\frac{\partial \varphi_{i}^{T}}{\partial \theta_{k}}={\left(\frac{\partial \varphi_{i}}{\partial \theta_{k}}\right)}^{T}$, similarly, $\frac{\partial \varphi_{i}^{T}}{\partial \alpha_{j}}={\left(\frac{\partial \varphi_{i}}{\partial \alpha_{j}}\right)}^{T}$.

Hence, Equations (33) and (34) can be rewritten as follows:

(35) $$\frac{\partial S_{\lambda }^{s}}{\partial \alpha_{j}}=S_{\lambda }^{s}[\frac{\partial \varphi_{i}}{\partial \alpha_{j}}\frac{1}{\varphi_{i}}+{\left(\frac{\partial \varphi_{i}}{\partial \alpha_{j}}\right)}^{T}\frac{1}{\varphi_{i}^{T}}]$$

(36) $$\frac{\partial S_{\lambda }^{bc}}{\partial \theta_{k}}=S_{\lambda }^{bc}[\frac{\partial \varphi_{i}}{\partial \theta_{k}}\frac{1}{\varphi_{i}}+{\left(\frac{\partial \varphi_{i}}{\partial \theta_{k}}\right)}^{T}\frac{1}{\varphi_{i}^{T}}]$$

Since $\frac{\partial S_{\lambda }^{s}}{\partial \alpha_{j}}=\frac{\partial S_{\lambda }^{bc}}{\partial \theta_{k}}=\frac{\partial^{2}\lambda_{i}}{\partial \theta_{k}\partial \alpha_{j}}$, add Equations (35) and (36), then the sensitivity coefficient can be obtained as follows:

(37) $$\frac{\partial^{2}\lambda_{i}}{\partial \theta_{k}\partial \alpha_{j}}=-\frac{1}{2}\{\varphi_{i}K_{k}^{s}\varphi_{i}^{T}[\frac{\partial \varphi_{i}}{\partial \alpha_{j}}\frac{1}{\varphi_{i}}+{\left(\frac{\partial \varphi_{i}}{\partial \alpha_{j}}\right)}^{T}\frac{1}{\varphi_{i}^{T}}]+\varphi_{i}K_{j}^{bc}\varphi_{i}^{T}[\frac{\partial \varphi_{i}}{\partial \theta_{k}}\frac{1}{\varphi_{i}}+{\left(\frac{\partial \varphi_{i}}{\partial \theta_{k}}\right)}^{T}\frac{1}{\varphi_{i}^{T}}]\}$$

### 2.2. Objective Function Based on Regularization Technology

Regularization technology, as an excellent tool, has been widely applied in solving the problem of underdetermined equation in damage identification [10]. Assuming the change of modal parameters caused by damage is linear, it can be expressed as:

(38) Sx=Δλ

where S denotes the sensitivity coefficient matrix, which can be calculated according to Equation (37); $x={\left[\theta ,\alpha \right]}^{T}$ is the combination of the stiffness reduction vectors of elemental damage and bearing damage; Δλ means the change of eigenvalue. Because of only a small number of damaged elements and bearings, x will be a sparse vector. Meanwhile, to consider the limitation of sensors and incomplete modal parameters, the dimension of Δλ is far less than x, which indicates the equation is underdetermined. Therefore, in order to obtain the expected sparsest solution, Equation (38) can be transformed into the optimization problem as follows:

(39) $$\min{\left\|x\right\|}_{1},s.t.{\left\|Sx-\Delta \lambda \right\|}_{2}\leq \varepsilon$$

Noting $\Delta \lambda =\lambda^{e}-\lambda^{a}$, the superscript *e* and *a* represent the experimental and analytical eigenvalues, respectively.

The above equation can be rewritten as follow:

(40) $$\min{\left\|x\right\|}_{1},s.t.{\left\|\lambda (x)-\lambda^{e}\right\|}_{2}\leq \varepsilon$$

where $\lambda (x)=Sx+\lambda^{a}$; ε is the error tolerance.

Moreover, based on Equation (40), the following $l_{1}$ regularization can be obtained:

(41) $$obj={\left\|\lambda (x)-\lambda^{e}\right\|}_{2}^{2}+\mu {\left\|x\right\|}_{1}$$

where μ>0 represents the regularization parameter, which is determined using the L-curve approach [11] or calculated by the equation of $\mu =\sigma \sqrt{2\log(p)}$, in which p is the cardinality of S [35].

Considering the damage identification problem and obtained eigenvalues, Equation (41) is further written as:

(42) $$obj=\frac{1}{m}{\left[\frac{\lambda_{i}^{a}(x)-\lambda_{i}^{e}}{\lambda_{i}^{e}}\right]}^{2}+\frac{\mu }{n}{\left\|x\right\|}_{1}$$

where $\lambda_{i}^{a}$ and $\lambda_{i}^{e}$ are the *i*-th analytical and experimental eigenvalues, respectively; *m* means the considered number of modes; *n* represents the length of x.

### 2.3. Damage Location Based on MSEBI

In a structure, the damage of structural elements and bearings often exist simultaneously, which increases the difficulty of damage detection and the cost of optimization computation. Aiming to this problem, MSEBI is utilized to locate the locations of damaged elements and bearings.

The modal strain energy of the element and the bearing can be calculated as follows:

(43) $$mse_{i}^{e}=\frac{1}{2}{\left(\varphi_{i}^{e}\right)}^{T}k_{k}^{e}\varphi_{i}^{e},k=1,2,\cdots ,nele,i=1,2,\cdots ,nm$$

(44) $$mse_{i,j}^{bc}=\frac{1}{2}{\left(\varphi_{i}^{bc}\right)}^{T}k_{j}^{bc}\varphi_{i}^{bc},j=1,2,\cdots ,nb,i=1,2,\cdots ,nm$$

where $\varphi_{i}^{e}$ and $\varphi_{i}^{bc}$ are the nodal displacement vectors of the *k*-th element and *j*-th bearing corresponding to the *i*-th mode shape, respectively; $k_{k}^{e}$ and $k_{j}^{bc}$ stand for the *k*-th elemental stiffness matrix and the *j*-th bearing stiffness matrix, respectively; *nm* represents the mode order considered; *nele* and *nb* are the total numbers of structural elements and bearings, respectively.

According to the above equations, the *i*-th total modal strain energy of the structure and bearings can be calculated as follows:

(45) $$mse_{i}=\sum_{e=1}^{nele}mse_{i}^{e},i=1,2,\cdots ,nm$$

(46) $$mse_{i}^{bc}=\sum_{j=1}^{nb}mse_{i,j}^{bc},i=1,2,\cdots ,nm$$

For the convenience of calculation, the normalized *i*-th modal strain energy of element and bearing can be defined as follows:

(47) $$nmse_{i}^{e}=\frac{mse_{i}^{e}}{mse_{i}}$$

(48) $$nmse_{i}^{bc}=\frac{mse_{i,j}^{bc}}{mse_{i}^{bc}}$$

Then taking the mean value of first *nm*-order normalized modal strain energy of element and bearing, Equations (47) and (48) can be rewritten as follows:

(49) $$mnmse^{e}=\frac{\sum_{i=1}^{nm}nmse_{i}^{e}}{nm},e=1,2,\cdots ,nele$$

(50) $$mnmse_{j}^{bc}=\frac{\sum_{i=1}^{nm}nmse_{i,j}^{bc}}{nm},j=1,2,\cdots ,nb$$

Thus, MSEBI can be obtained as:

(51) $$MSEBI^{e}=\max\left[0,\frac{{\left(mnmse^{e}\right)}^{E}-{\left(mnmse^{e}\right)}^{A}}{{\left(mnmse^{e}\right)}^{A}}\right],e=1,2,\cdots ,nele$$

(52) $$MSEBI_{j}^{bc}=\max\left[0,\frac{{\left(mnmse_{j}^{bc}\right)}^{E}-{\left(mnmse_{j}^{bc}\right)}^{A}}{{\left(mnmse_{j}^{bc}\right)}^{A}}\right],j=1,2,\cdots ,nb$$

where max[] represents the action of taking the maximum value; the superscripts of E and A mean experimental and analytical respectively; when the analytical and experimental modal strain energy are the same, MSEBI = 0, namely, the element or bearing is intact, otherwise, the damage may occur (MSEBI > 0).

## 3. Moth-Flame-Invasive Weed Optimization

### 3.1. Moth-Flame Optimization

Moth-flame optimization (MFO), as a novel optimization tool, was proposed by Mirjalili [36]. The inspiration of MFO can be traced back to the transverse orientation of moths at night, but it was developed by the approach of a moth flying around the flame or candle. The basic theory of MFO can be explained that some individuals of the moth with an attribute of position are first initialized randomly in a *D*-dimensional solution space:

(53) $$M_{i}=(m_{i1},m_{i2},\cdots ,m_{ij}),i=1,2,\cdots ,n;j=1,2,\cdots ,D$$

where *n* denotes the number of moths; *D* represents the dimension of optimization problem. At the same time, the artificial light, namely, flame, will be marked as follows:

(54) $$Flame_{i}=(Flame_{i1},Flame_{i2},\cdots ,Flame_{ij}),i=1,2,\cdots ,n;j=1,2,\cdots ,D$$

The fitness values of each moth individual and flame are stored in two vectors, which can be shown as follows:

(55) $$OM={\left[OM_{i}^{it}\right]}^{T},i=1,2,\cdots ,n$$

(56) $$OF={\left[OF_{i}^{it}\right]}^{T},i=1,2,\cdots ,n$$

where OM and OF represent the fitness value vectors of moth individual and flame, respectively; *it* means the current number of the iteration. Then, the logarithmic spiral function is utilized to update the position of each moth:

(57) $$S(M_{i},Flame_{j})=D_{i}\cdot e^{bt}\cdot \cos(2\pi t)+Flame_{j}$$

where $D_{i}$ stands for the spatial distance from *i*-th moth to *j*-th flame, the constant *b* is a factor to define the spiral shape function, and *t* is a random number between −1 and 1.

Furthermore, the adaptive decrease mechanism of flames is incorporated into optimization algorithm to ensure the powerful exploitation of optimization process, which also keeps the moth individual always flying around the optimal solution from the first iteration to iteration termination. The mathematical formula of the mechanism can be written as follows:

(58) $$Fn^{it}=round(Fn_{\max}-Iteration\cdot \frac{Fn_{\max}-1}{Iteration_{\max}})$$

where $Fn^{it}$ and $Fn_{\max}$ are the flame number of the *it*-th iteration and the max number of flame; Iteration stands for the current count of iteration; $Iteration_{\max}$ means the maximum number of iteration, and round() denotes the action of taking the integer portion.

### 3.2. Invasive Weed Optimization

Invasive weed optimization (IWO) is inspired by the situation of colonization of invasive weeds [37,38]. The colonizing behavior of weeds can be described in the formalization language of mathematics, namely, some weeds are randomly generated in the *D*-dimensional problem space with the characteristic of the position:

(59) $$W_{i}=(w_{i1},w_{i2},\cdots ,w_{ij}),i=1,2,\cdots ,n;j=1,2,\cdots ,D$$

where *n* denotes the initial number of weed; *D* represents the dimension of the problem. Then, based on the fitness of each weed, the seed of each one is calculated by the equation as follows:

(60) $$\omega_{i}=\frac{f(w_{i})-f_{\min}}{f_{\max}-f_{\min}}(s_{\max}-s_{\min})+s_{\min}$$

where $f(w_{i})$ means the fitness value of *i*-th weed; $f_{\min}$ and $f_{\max}$ are the worst and best fitness in the current population, respectively; $s_{\min}$ and $s_{\max}$ represent the minimum and maximum seed number that one weed can produce, respectively.

Subsequently, the produced seeds are spread over the search space using a normally distributed random number, whose standard deviation at current iteration can be expressed by:

(61) $$\sigma_{iteration}=\frac{{\left(Iteration_{\max}-Iteration\right)}^{n}}{{\left(Iteration_{\max}\right)}^{n}}(\sigma_{initial}-\sigma_{final})+\sigma_{final}$$

where Iteration stands for the current count of iteration; $Iteration_{\max}$ means the maximum number of iteration; $\sigma_{initial}$ and $\sigma_{final}$ represent the initial and final values of standard deviation, respectively; n=3 means the nonlinear modulation coefficient. Thus, the position of weeds can be updated by the equation as follows:

(62) $$W_{i}^{it+1}=W_{i}^{it}+\sigma_{iteration}\cdot rand()$$

Furthermore, the principle of the growing competition is adopted in the algorithm, and it can be described that while evaluating the fitness of all weeds and seeds, the poor weeds and/or seeds are eliminated to reach the maximum number of preset populations.

### 3.3. Chaotic Moth-Flame-Invasive Weed Optimization Hybrid Algorithm

MFO is of powerful local searching ability, but it is unable to ensure the performance in global search, especially for the diversity of moth individuals in the late iterations is poor. Therefore, a hybrid algorithm, chaotic moth-flame-invasive weed optimization hybrid algorithm (CMF-IWO), is proposed to obtain a better optimization result. In the hybrid algorithm, the mechanism of seed spreading and growing competition are incorporated into MFO, meanwhile, the population initialization approach of reverse learning [39], chaos theory [40], and evolutional strategy are also adopted to enhance the diversity of the population.

**Figure 1:**
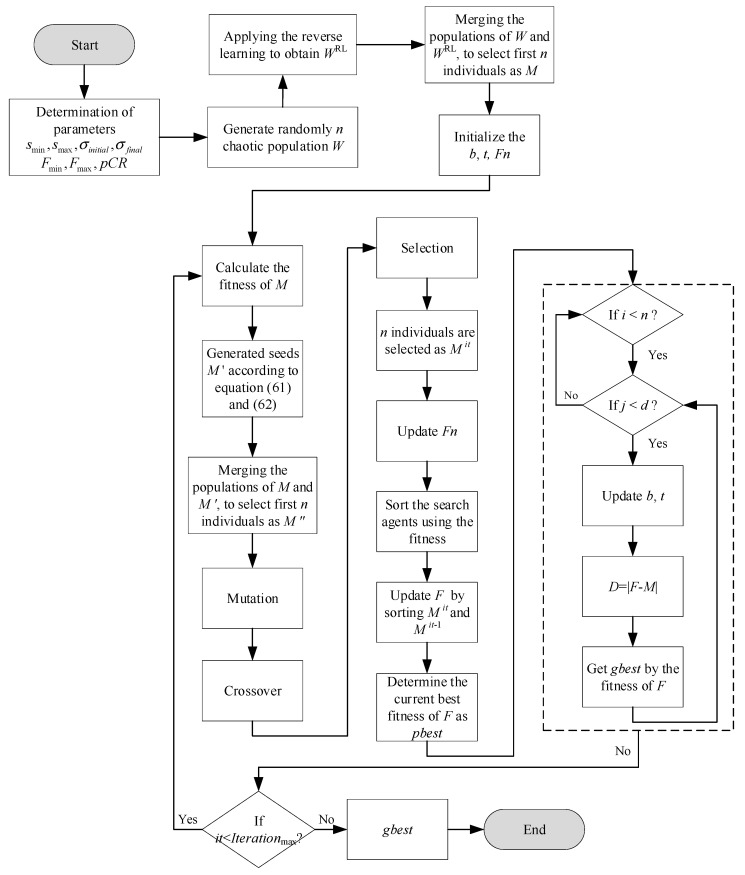
The flowchart of CMF-IWO hybrid algorithm.

The flowchart of CMF-IWO is shown in Figure 1.

The basic procedures of CMF-IWO can be summarized as follows:

(1) To initialize the chaotic populations of *n* based on the chaos theory, the related equation can be defined as follows:
  (63) $$w_{i}=lb+\xi_{i}\cdot (ub-lb)$$
  where lb and ub are the low and upper bounds respectively; $\xi_{i}$ denotes the chaotic vector, which can be generated by the logistic chaos mapping:
  (64) $$\xi_{i+1}=u\cdot \xi_{i}\cdot (1-\xi_{i})$$
  where u is a scalar, when u=4, the system is in chaos.
(2) To apply the operation of reverse learning, the individuals of reverse learning can be produced as follows:
  (65) $$w_{i}^{RL}=lb+ub-w_{i}$$
  Then merge $w_{i}$ and $w_{i}^{RL}$, and the fitness of each individual is evaluated. After that, according to the ranking of the fitness, the first *n* weeds are selected as moth individuals to be input into MFO.
(3) In MFO, the moth individuals are spread over the search space according to Equation (61) to enhance the diversity, and the evolutional strategy is also incorporated in this stage. The operation of mutation can be described as follows:
  (66) $$m_{i}=m_{ij}+F(m_{ik}-m_{ir})$$
  where $F=(F_{\max}-F_{\min})*rand$ is the scale factor, $m_{ij}$, $m_{ik}$, and $m_{ir}$ are the *j*-th, *k*-th, and *r*-th element of *i*-th moth, respectively. The operations of crossover and selection can be defined as:
  (67) $$m_{ij}=\{\begin{array}{c} m_{ij}^{1}\;rand(0,1)\leq pCR\\ m_{ij}^{2}\;rand(0,1)>pCR \end{array}$$
  (68) $$m_{i}=\{\begin{array}{c} m_{i}^{1}\;f(m_{i}^{1})\leq f(m_{i}^{2})\\ m_{i}^{2}\;f(m_{i}^{1})>f(m_{i}^{2}) \end{array}$$
  where the pCR represents the probability of crossover.
(4) Based on the obtained individuals of the previous step, the first *n* moth individuals are selected according to their fitness values. Then the remaining steps of MFO are conducted to obtain the optimization results.

### 3.4. Evaluation of the Proposed Algorithm

#### 3.4.1. Evaluation Using Benchmark Functions

**Figure 2:**
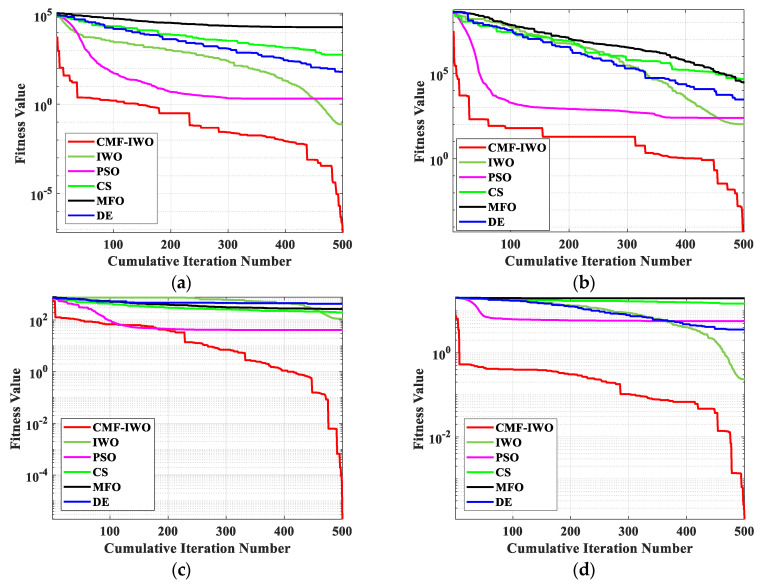

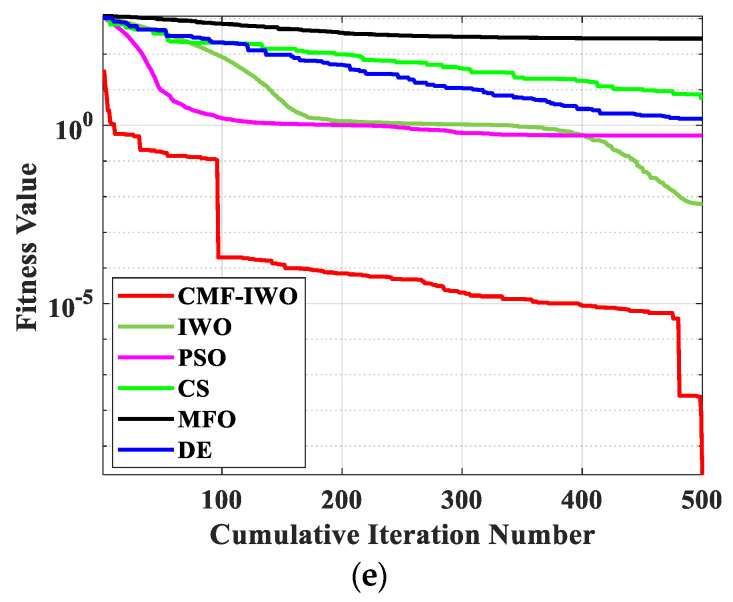
The average iterative curves of benchmark functions: (**a**) F1; (**b**) F2; (**c**) F3; (**d**) F4, and (**e**) F5.

The optimization ability and computational accuracy of the hybrid algorithm are first evaluated with five mathematical benchmark functions (Table 1) and compared with existing optimization algorithms, such as MFO, IWO, Particle Swarm Optimization (PSO), Cuckoo Search (CS), and Differential Evolution (DE). The parameter settings of each algorithm are listed in Table 2, and each algorithm is performed 50 times with maximum iterations of 500 and maximum populations of 100, the average results and iterative curves are shown in Table 3 and Figure 2.

From Table 3, it can be seen that the proposed hybrid algorithm can achieve better optimal results in the optimizations of five benchmark functions, which can be owed to the chaotic population, and the mechanism of seed space spreading can enhance the diversity of the initial population. Meanwhile, the operation of reverse learning can obtain elite populations. These two improvements can guarantee that the initial populations are of high quality and diversity. In addition, the iterative curves of Figure 2 indicate that the curves of CMF-IWO are steeper than those of other algorithms, which demonstrates that the convergence speed of CMF-IWO is good. At the same time, compared to the other algorithms, the proposed algorithm can escape from local optimal, whose main reason may be the fusion of evolutional strategy.

#### 3.4.2. Evaluation Using Numerical Example of Structural Damage Identification

**Figure 3:**
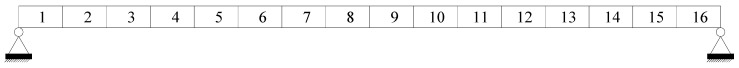
The simply supported beam.

In order to further assess the optimization ability of the proposed hybrid algorithm, a simply supported beam with 16 elements is exploited (Figure 3). For the beam, its Young’s modulus is 3.0 × 10^10^ Pa, the mass density is 2450 kg/m^3^, the cross-sectional area is 0.05 m^2^, the inertia moment is 4.16 × 10^−5^ m^4^, the length of each element is 0.5 m. Three damage cases, including single-point damage, double-point damage, and multiple-point damage, are introduced by the reduction of the stiffness, the details are listed in Table 4.

**Figure 4:**
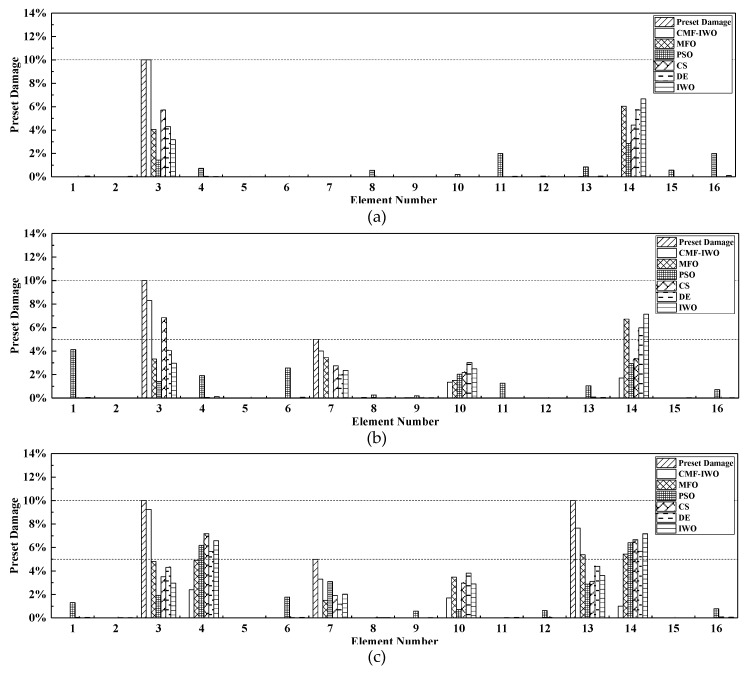
Damage identification results of simply supported beam: (**a**) Case 1; (**b**) Case 2, and (**c**) Case 3.

Because of the beam with the simply supported boundary conditions, namely, it has no bearings on each side. The bearing damage factor $\alpha_{j}$ is defined as zeros, only the elemental damage factor $\theta_{k}$ is considered. The parameters setting of six algorithms are the same as Table 2, each algorithm is performed seven times with maximum iteration of 500 and maximum population of 100, the average damage identification results are illustrated in Figure 4.

As shown in Figure 4, for the single-point damage, CMF-IWO can determine the damage location and quantify the damage severity with a higher accuracy. However, other algorithms cannot achieve satisfactory results. Then in Case 2, some errors occur in all the six algorithms, the main reason is that the natural frequencies do not contain the spatial information of structural damage, so it is not easy to obtain very precise results by using merely frequencies. However, comparatively, the error of the proposed algorithm is less than the other five algorithms, which indicates the optimization performance of CMF-IWO is better than other algorithms. Furthermore, the same conclusion can be obtained in Case 3.

To summarize, with limited modal characteristics, by using the proposed CMF-IWO hybrid algorithm, better damage identification results can be achieved than the other five algorithms, which has better potential in structural damage detection.

### 3.5. Damage Detection Methodology

Based on the proposed regularization objective function and CMF-IWO hybrid algorithm, a novel damage detection approach is put forward, which can identify the damage of bearings, as well as the damage of structural elements. The main steps of this method can be summarized as follows:

(1) MSEBI of structural elements and bearings is calculated and used to detect the locations of damage;
(2) According to the detected damage location, the sensitivity coefficients of the suspected structural elements and bearings are calculated, which are adopted to construct the regularization objective function;
(3) The regularization objective function constructed in the previous step is input into CMF-IWO hybrid algorithm to quantify the damage severities of structural elements and bearings.

## 4. Damage Detection Examples

### 4.1. Numerical Study

**Figure 5:**
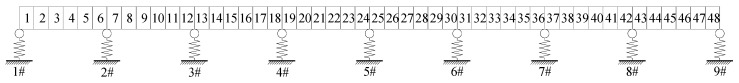
The 8-span continuous beam.

In this section, as shown in Figure 5, an 8-span continuous beam with 48 elements and 9 bearings is introduced to verify the proposed damage identification method. The length and cross-sectional area of each element are 0.5 m and 0.03 m^2^, respectively, its material properties, such as Young’s modulus, mass density, and inertia moment are 3.45 × 10^10^ Pa, 2500 kg/m^3^, and 2.5 × 10^−5^ m^4^, respectively. The vertical stiffness of the bearings is 1.0 × 10^6^ KN/m. The damage of structural elements and bearings are both simulated using reduction of stiffness [41,42,43,44]. Five damage cases are introduced, which are shown in Table 5.

**Figure 6:**
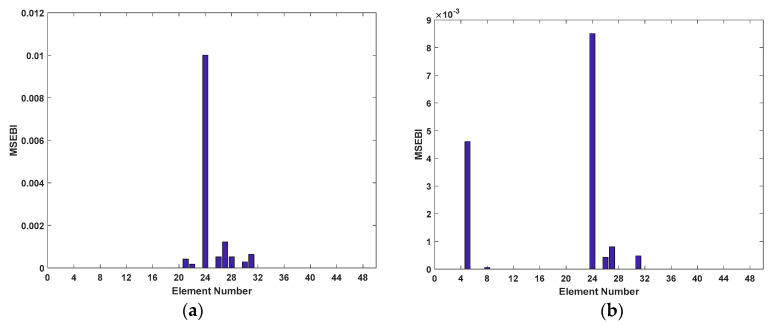

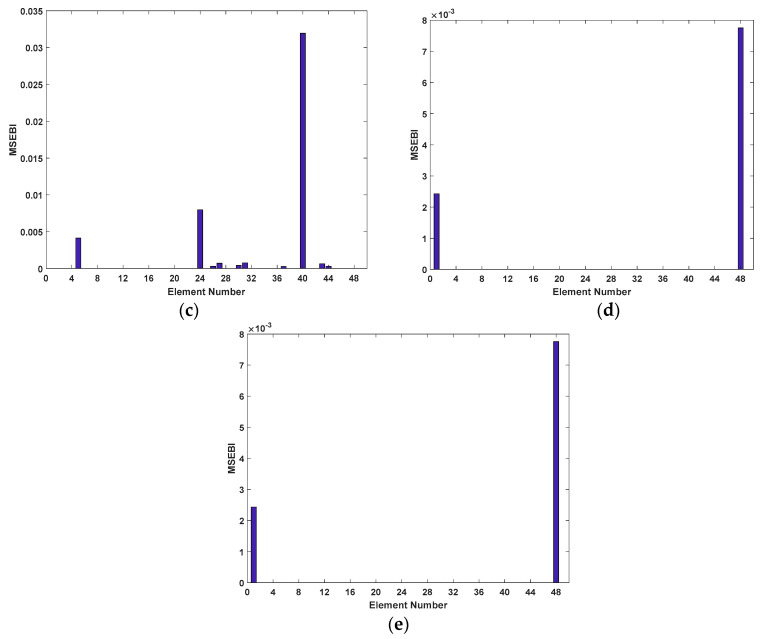
Results of structural elements damage location: (**a**) Case 1; (**b**) Case 2; (**c**) Case 3; (**d**) Case 4; and (**e**) Case 5.

**Figure 7:**
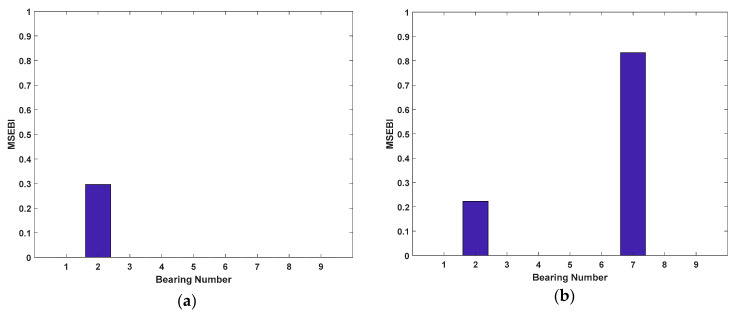

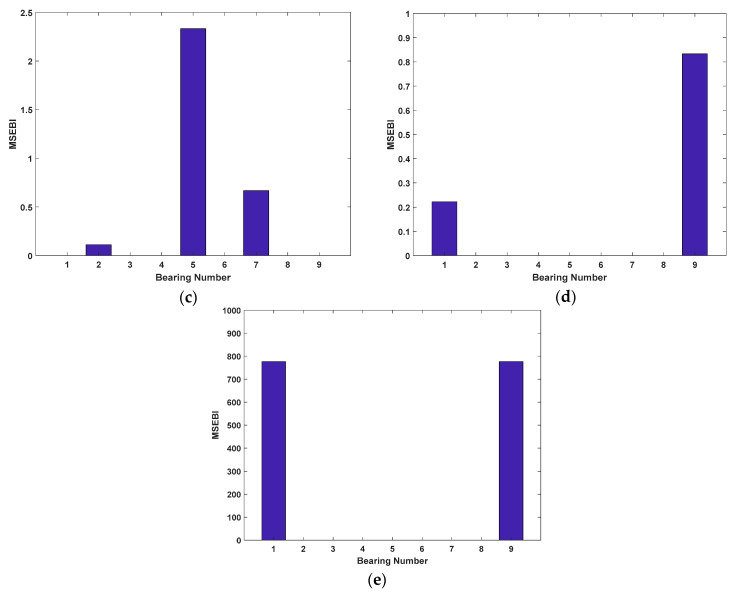
Results of structural bearings damage location: (**a**) Case 1; (**b**) Case 2; (**c**) Case 3; (**d**) Case 4; and (**e**) Case 5.

The first six modal parameters are exploited to detect the damage of structural elements and bearings. Also, the maximum iterations, populations, and parameters setting are the same as Section 3.4. The results of the damage location are depicted in Figure 6 and Figure 7.

As shown in Figure 6, the suspected damaged elements of the five damage cases are detected accurately. Also, in Cases 1 to 3, there still exist some elements whose identified damage are not zero, but compared to the damaged elements, the values are so small that they can be ignored. Hence, it is revealed that the suspected damaged elements for Cases 1 to 3 can be reduced from 48 to 1, 2, and 3 elements respectively. On the other hand, from Figure 7, it is clearly observed that MSEBI can locate the locations of damaged bearings with high accuracy. To summarize, MSEBI not only can detect the damage location of structural elements but also identify the locations of damaged bearings.

**Figure 8:**
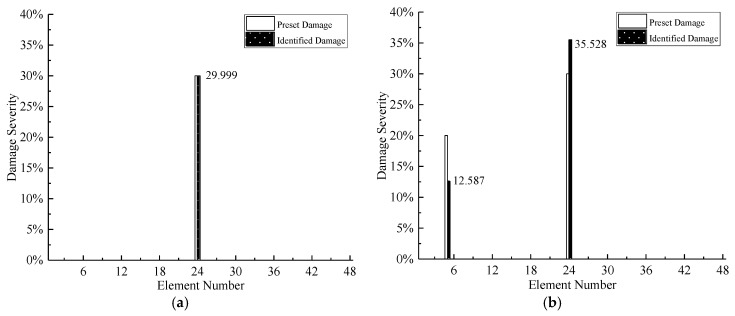

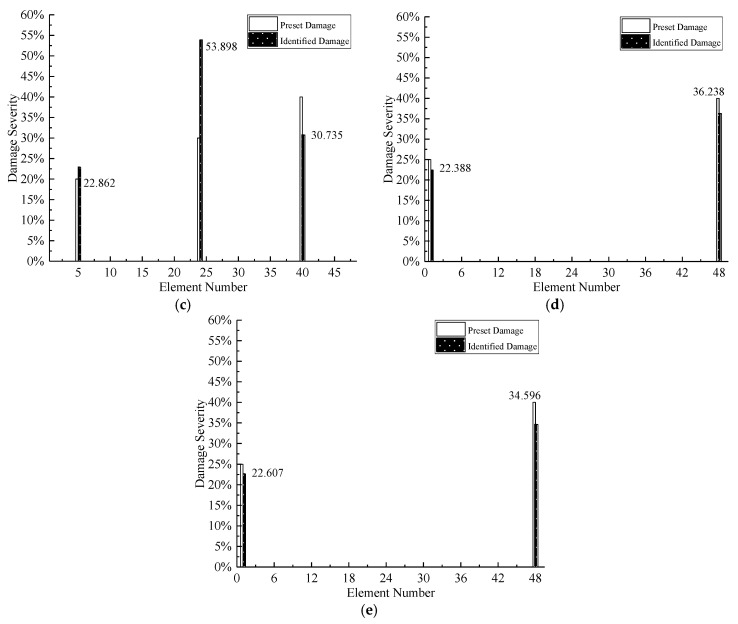
Damage Detection Results of Structural Elements: (**a**) Case 1; (**b**) Case 2; (**c**) Case 3; (**d**) Case 4 and (**e**) Case 5.

**Figure 9:**
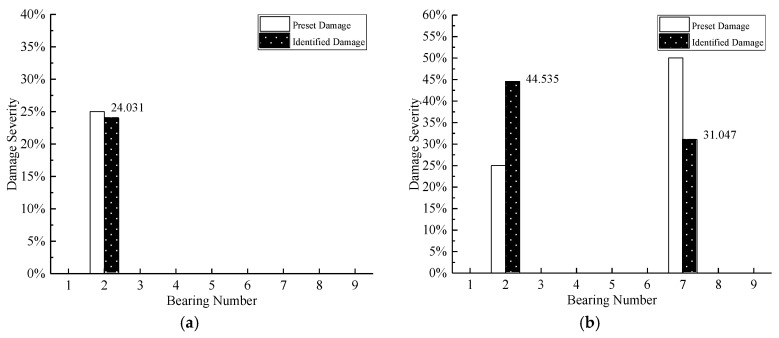

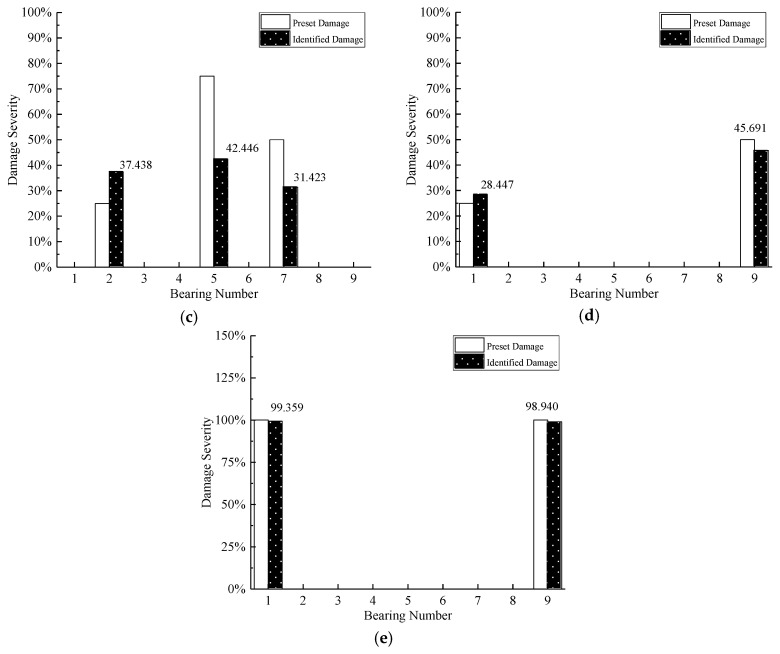
Damage detection results of bearings: (**a**) Case 1; (**b**) Case 2; (**c**) Case 3; (**d**) Case 4; and (**e**) Case 5.

Because modal parameters are more sensitive to the damage of structural elements than the damage of bearings, if the damage severities of elements and bearings are simultaneously determined in the process of damage detection, the change of modal parameters caused by the damage of bearings will be masked by elemental damage. Thus, at first, assuming the bearings are intact, the damage severity of structural elements is determined. After that, the determined elemental damage condition is input to the procedure of damage detection, and the damage severity of bearing is quantified. The average results of seven times iterations are demonstrated in Figure 8 and Figure 9.

As shown in Figure 8, because of the location operation of MSEBI, the proposed method can detect the damaged elements with high accuracy. However, regarding the quantification of damage severity, the performance is not very satisfactory. Only for Case 1, the result is precise, different degrees of error occur in all the other cases, this phenomenon can be explained as follows: (1) The eigenvalues do not include the spatial information of structural damage, which cannot obtain the accurate detection results; (2) the existence of bearings damage may apply some adverse influence on the identification performance.

The detection results of bearings damage are shown in Figure 9, it can be observed that the damage of Case 1, Case 4, and Case 5 can be quantified with satisfactory precision. However, there are different errors in other damage cases, such as damage severity shifting for Case 2 and inaccurate quantification results for Case 3. The reason also can be owed to the missing spatial information of eigenvalues, at the same time, the previous damage identification results of structural elements are the key factors to the damage detection of bearings. On the other hand, comparing Case 2, Case 4, and Case 5, it can be found that the more serious the damage severity of bearings, the more severe the fluctuations of the eigenvalues. Meanwhile, the comparison also indicates that the proposed method is more suitable to detect the serious damage of bearings, like bearing separation.

### 4.2. Experimental Example

**Figure 10:**
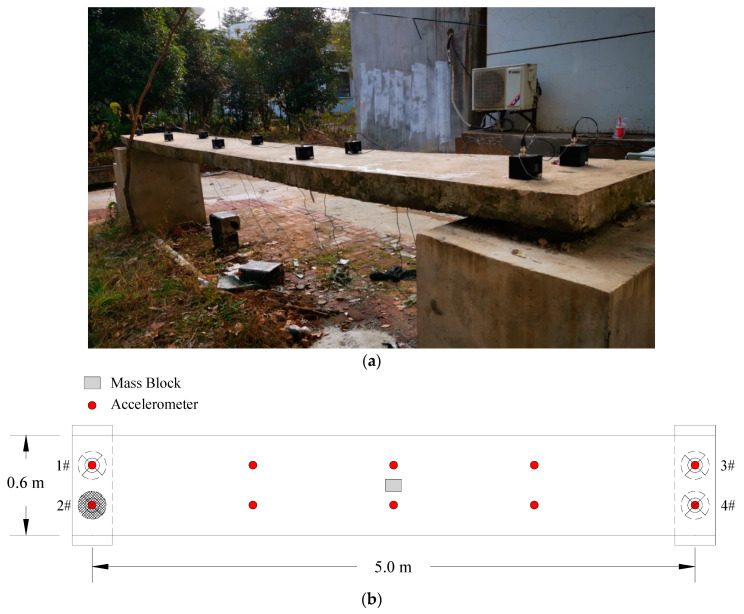
The simply supported reinforced concrete plate: (**a**) view of modal test; (**b**) layout of sensors and mass block.

In this section, an experimental example of a simply supported reinforced concrete plate is adopted to further assess the proposed damage detection approach. As shown in Figure 10a, the plate is located in the campus of Wuhan Institution of Technology; its measured size is 5.4 m × 0.6 m × 0.12 m (length × width × thickness), with the Young’s modulus of 3 × 10^10^ Pa, the mass density of 2410 kg/m^3^. Meanwhile, there were four rubber bearings placed on the two ends to support the plate. Additionally, the lengths of both the overhangs were 0.2 m at the two ends. In the natural environment, irregular hammer excitation was conducted to make the plate vibrate, ten accelerometers were installed on the top surface of the plate to collect the signal of acceleration. The layout of sensors can be seen as Figure 10b.

**Figure 11:**
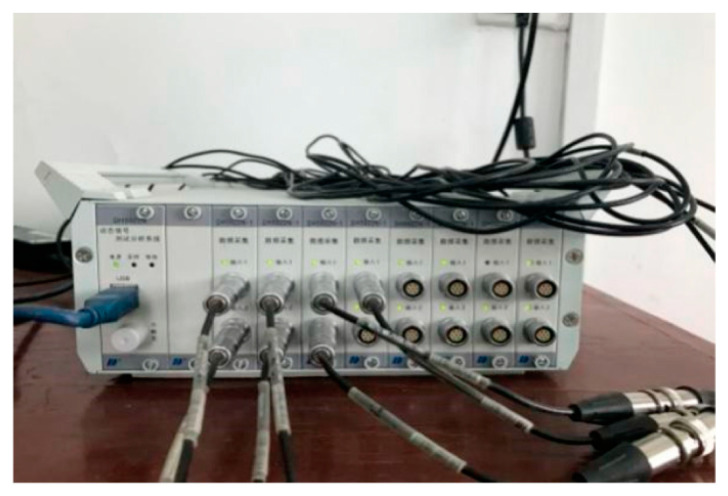
DH5922 vibration testing system

The acceleration data were captured by a DH5922 vibration testing system (Figure 11) which has the advantages of light mass and convenient use etc. The operation temperature of acceleration acquisition instrument ranges from 0 °C to 60 °C and it is also a universal dynamic signal test and analysis system which can complete the testing and analysis of stress, strain, vibration, shock, etc. The instrument has 16 24-bit IEPE input channels that are equipped with an anti-mixing filter, and supports sampling frequency up to 51.2 k Hz. The system was connected with the acceleration sensor by L5 coaxial extension wire and placed in the center of the equal dividing line to collect the acceleration signal. The operation temperature of acceleration sensor ranged from −40 °C to 80 °C and its tolerance was ±1%.

**Figure 12:**
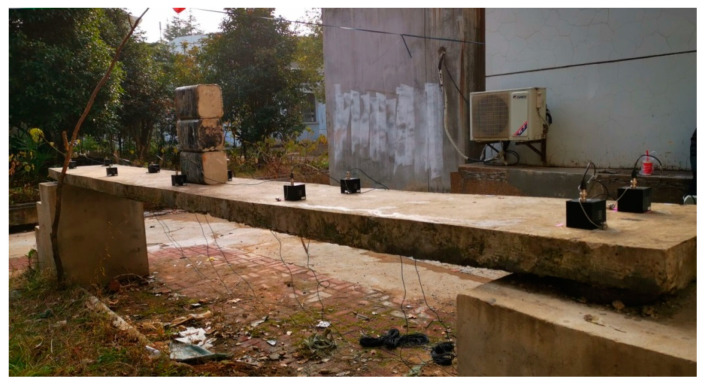
The simply supported reinforced concrete plate with additional mass blocks.

Meanwhile, in the experiment, because of its difficulties in introducing the stiffness change of concrete plate, the damage was simulated by the approach of applying additional mass block with a length of 0.3 m, width of 0.2 m, and thickness of 0.15 m (Figure 12); moreover, the placed location was the center of the plate. Also, bearing 2# was removed to simulate the common disease, namely, bearing separation. Eight cases were set, and corresponding modal tests were conducted, the details of each case and the measured natural frequencies are listed in Table 6 and Table 7, respectively.

**Figure 13:**
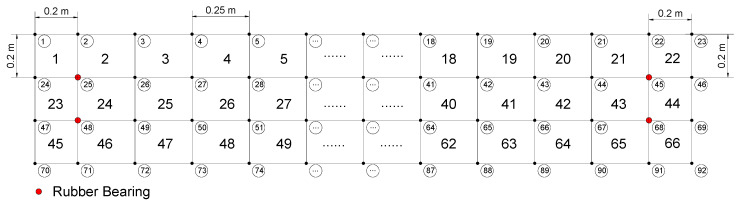
The numbering diagram of elements and nodes.

Then, MATLAB is used to construct the finite element model of the plate. The plate is meshed to 66 elements, which is modeled by 20-node shell element; furthermore, the rubber bearings were simulated by the 3-D spring elements. The numbering and meshing diagram of elements and nodes are depicted in Figure 13. Because of the limitation of sensors, the modal shapes were incomplete, meanwhile, the structure is 3-dimensional, hence it is difficult to calculate the modal assurance criterion, thus, only the natural frequencies are adopted. The analytical and experimental natural frequencies are extracted and listed in Table 8.

As shown in Table 8, there exist some errors between the actual structure and the finite element model, especially for the higher-order modes, which can be owed to the environmental effects, instrument errors, and size deviations of the model. Thus, model updating is conducted using the CMF-IWO hybrid algorithm. After model updating, it can be revealed that the consistency between analytical and experimental models is really good, which means the model can be used for the baseline model of damage detection.

Assuming the mass of the plate is uniformly distributed, that is to say, when the size is the same, the mass of each element is equal. By introducing the mass additional factors, the total mass can be calculated as:

(69) $$M^{Mass}=\sum_{i=1}^{nele}(1+\beta_{i})m_{i}$$

where $M^{Mass}$ represents the total weight of the plate and additional mass blocks; $m_{i}$ and $\beta_{i}$ are the *i*-th elemental mass and additional factor respectively. Hence, when $\beta_{i}$ is obtained, the weight of the additional mass block can be identified based on elemental mass.

However, because of the limitation of sensors, the measured mode shapes are incomplete resulting in the fact that MSEBI cannot be obtained. For the purpose of bearings damage and additional mass detection, the location of mass blocks is assumed to be known, thus the search range is reduced. Additionally, the detection of mass and bearings damage are separated, namely, the mass change is first determined, after that the damage of the bearings is identified. According to Equation (42), the objective function can be defined as follows:

(70) $$obj=\frac{1}{m}{\left[\frac{\lambda_{i}^{a}(X)-\lambda_{i}^{e}}{\lambda_{i}^{e}}\right]}^{2}+\frac{\mu }{n}{\left\|X\right\|}_{1}$$

where X=[β,α]. For the Cases 2–8, the detection procedure is carried out for seven times, the average identification results are extracted and depicted in Table 9.

As listed in Table 9, the proposed method can precisely detect the separation of bearings. However, for the identification of mass change, the errors occur, namely, the inaccurate weight quantification. For Cases 3 and 6, the identified results are acceptable, but for other cases, the overestimating or underestimating has emerged, which may be attributed to the inaccurate measured data and the errors of the finite element model.

## 5. Conclusions

A novel bearings damage detection method using sensitivity analysis and chaotic moth-flame-invasive weed optimization hybrid algorithm has been put forward to determine the damage of structural elements and bearings. According to the obtained results, some conclusions and prospects can be summarized as follows:

(1) The sensitivity coefficients of eigenvalues to the damage factors of structural elements and bearings provide a good evaluation approach to research the influences of damage of structural elements and bearings to eigenvalues, meanwhile, which is the basis for constructing the regularization objective function.
(2) MSEBI, as a damage location index, is able to accurately detect the damage location of structural elements as well as identify the locations of damaged bearings with high precision. At the same time, this damage location approach can greatly reduce the search range of damage detection and promote detection effectiveness.
(3) Compared to PSO, CS, MFO, DE, and IWO, the proposed hybrid algorithm, CMF-IWO, is demonstrated with good convergence speed and global search performance, which is of good potential for overcoming the problem of local optimal in damage detection.
(4) The proposed method is proved to be well applied in the bearings damage detection of numerical simulation, supported by the study case. Compared to the existing methods, the proposed method is easy and convenient to conduct and only the first few modal characteristics are needed. Hence complex calculation can be avoided. However, because of some uncertain factors and errors, such as the inaccuracy of instrument measurement, incomplete modal information, inevitable environmental noise, and the deviation of the finite element model, there still exist some difficulties to obtain very precise results in practical application.

## Figures and Tables

**Table 1:** The benchmark functions.

Function	Definition	Dimension	Range
F1	$f_{1}(x)=\sum_{i=1}^{D}x_{i}^{2}$	*D* = 50	[−100,100]
F2	$f_{2}(x)=\sum_{i=1}^{D}\left[100{\left(x_{i+1}-x_{i}^{2}\right)}^{2}+{\left(x_{i}-1\right)}^{2}\right]$	*D* = 50	[−30,30]
F3	$f_{3}(x)=\sum_{i=1}^{D}\left[x_{i}^{2}-10\cos(2\pi x_{i})+10\right]$	*D* = 50	[−5.12,5.12]
F4	$f_{4}(x)=-20\exp(-0.2\sqrt{\frac{1}{n}\sum_{i=1}^{D}x_{i}^{2}})-\exp(\frac{1}{n}\sum_{i=1}^{D}\cos(2\pi x_{i}))+20+e$	*D* = 50	[−32,32]
F5	$f_{5}(x)=\frac{1}{4000}\sum_{i=1}^{D}x_{i}^{2}-\prod_{i=1}^{D}\cos(\frac{x_{i}}{\sqrt{i}})+1$	*D* = 50	[−600,600]

**Table 2:** The parameters settings of each algorithm.

Algorithm	Parameters
CMF-IWO	$s_{\max}=15$, $s_{\min}=0$, $\sigma_{initial}=10$, $\sigma_{final}=0.00001$, pCR=0.9, $F_{\min}=0.2$, $F_{\max}=0.8$
MFO	/
IWO	$s_{\max}=15$, $s_{\min}=0$, $\sigma_{initial}=10$, $\sigma_{final}=0.00001$, initial population: 25
PSO	$c_{1}=c_{2}=2$, $\omega_{\max}=0.9$, $\omega_{\min}=0.4$
CS	$P_{a}=0.25$
DE	pCR=0.9, $F_{\min}=0.2$, $F_{\max}=0.8$

**Table 3:** Statistical results of five benchmark functions.

Function	Parameters	Best	Worst	Mean	Std
F1	CMF-IWO	1.0887 × 10^−9^	2.5681 × 10^−7^	6.7496 × 10^−8^	6.4762 × 10^−8^
	MFO	52.9446	2.0310 × 10^4^	5851.1526	6630.1173
	IWO	0.0523	0.1041	0.0773	0.0130
	PSO	0.7630	2.8517	1.6985	0.5147
	CS	329.5988	818.0726	588.6970	117.3877
	DE	27.3037	129.5271	67.8251	25.0673
F2	CMF-IWO	6.7661 × 10^−8^	6.4539 × 10^−5^	1.6294 × 10^−5^	1.8073 × 10^−5^
	MFO	1.5441 × 10^4^	7.9961 × 10^7^	1.7465 × 10^6^	1.1287 × 10^7^
	IWO	53.6460	2250.7460	323.9226	453.1753
	PSO	81.8194	402.8715	194.5452	77.3596
	CS	2.5492 × 10^4^	9.0476 × 10^4^	5.6018 × 10^4^	1.6313 × 10^4^
	DE	2396.3380	2.4560 × 10^4^	7.1717 × 10^3^	4.029 × 10^3^
F3	CMF-IWO	2.9532 × 10^−8^	4.2936 × 10^−5^	1.1240 × 10^−5^	1.1753 × 10^−5^
	MFO	172.1081	453.5721	285.1176	53.0783
	IWO	73.1735	172.1583	121.4345	23.9014
	PSO	19.2279	60.4631	33.9154	9.0074
	CS	196.3107	286.7401	245.3998	19.5425
	DE	377.7231	462.2816	425.0265	16.5028
F4	CMF-IWO	1.6950 × 10^−5^	2.0 × 10^−4^	1.0 × 10^−4^	5.9477 × 10^−5^
	MFO	5.2021	19.9651	19.0605	2.8102
	IWO	0.2356	1.1460	0.5189	0.2578
	PSO	3.7516	6.0987	4.8038	0.6294
	CS	12.8120	17.7599	15.2283	1.2716
	DE	3.0005	19.9528	4.0701	2.7859
F5	CMF-IWO	9.3958 × 10^−11^	1.6634 × 10^−8^	3.3969 × 10^−9^	3.3064 × 10^−9^
	MFO	2.3160	273.2845	48.9034	58.2422
	IWO	0.0024	0.0254	0.0098	0.0054
	PSO	0.5038	0.9340	0.7803	0.09630
	CS	3.7573	8.9510	6.3427	1.1683
	DE	1.3510	2.4297	1.5854	0.1985

**Table 4:** Three damage cases.

Case	Damage Severity @ Element Number
1	10% @ 3
2	10% @ 3, 5% @ 7
3	10% @ 3, 5% @ 7, 10% @ 13

**Table 5:** Five damage cases.

Case	Damage Severity @ Element Number	Damage Severity @ Bearing Number
1	30% @ 24	25% @ 2#
2	20% @ 5, 30% @ 24	25% @ 2#, 50% @ 7#
3	20% @ 5, 30% @ 24, 40% @ 40	25% @ 2#, 75% @ 5#, 50% @ 7#
4	20% @ 1, 40% @ 48	25% @ 1#, 50% @ 9#
5	20% @ 1, 40% @ 48	99.9% @ 1#, 99.9% @ 9#

**Table 6:** Eight cases of the simply supported reinforced concrete plate.

Case	Mass/kg	Bearing
1	/	/
2	/	2#(removed)
3	20	/
4	20	2#(removed)
5	40	/
6	40	2#(removed)
7	60	/
8	60	2#(removed)

**Table 7:** The measured natural frequencies of eight cases.

Mode	Case 1	Case 2	Case 3	Case 4	Case 5	Case 6	Case 7	Case 8
1	7.28	7.357	7.18	7.042	6.874	6.850	6.743	6.688
2	27.314	26.734	26.827	26.318	26.46	26.418	25.837	26.095
3	60.054	59.479	58.709	59.351	56.903	57.348	56.408	54.380
4	103.218	103.097	102.913	104.520	102.084	103.957	102.039	102.105
5	153.48	153.454	153.204	153.439	150.728	151.203	147.903	153.554

**Table 8:** The analytical and experimental natural frequencies.

Mode	Experimental/Hz	Before Model Updating		After Model Updating	
		Analytical /Hz	Error /%	Analytical /Hz	Error/%
1	7.28	7.505	3.09	7.280	0.0006
2	27.314	26.791	1.91	27.315	0.0022
3	60.054	62.131	3.45	60.057	0.0049
4	103.218	89.243	13.53	103.225	0.0067
5	153.48	137.549	10.38	153.696	0.1406

**Table 9:** The identification results of additional mass and bearings damage.

Case	Identified Mass/kg	Identified Damage Severity @ Bearing Number
2	/	98.42% @ 2 #
3	23.008	/
4	9.045	98.41% @ 2 #
5	30.316	/
6	39.777	98.68% @ 2 #
7	72.353	/
8	76.201	98.35% @ 2 #
